# Do Bat Gantries and Underpasses Help Bats Cross Roads Safely?

**DOI:** 10.1371/journal.pone.0038775

**Published:** 2012-06-13

**Authors:** Anna Berthinussen, John Altringham

**Affiliations:** Institute of Integrative and Comparative Biology, University of Leeds, Leeds, United Kingdom; University of Western Ontario, Canada

## Abstract

Major roads can reduce bat abundance and diversity over considerable distances. To mitigate against these effects and comply with environmental law, many European countries install bridges, gantries or underpasses to make roads permeable and safer to cross. However, through lack of appropriate monitoring, there is little evidence to support their effectiveness. Three underpasses and four bat gantries were investigated in northern England. Echolocation call recordings and observations were used to determine the number of bats using underpasses in preference to crossing the road above, and the height at which bats crossed. At gantries, proximity to the gantry and height of crossing bats were measured. Data were compared to those from adjacent, severed commuting routes that had no crossing structure. At one underpass 96% of bats flew through it in preference to crossing the road. This underpass was located on a pre-construction commuting route that allowed bats to pass without changing flight height or direction. At two underpasses attempts to divert bats from their original commuting routes were unsuccessful and bats crossed the road at the height of passing vehicles. Underpasses have the potential to allow bats to cross roads safely if built on pre-construction commuting routes. Bat gantries were ineffective and used by a very small proportion of bats, even up to nine years after construction. Most bats near gantries crossed roads along severed, pre-construction commuting routes at heights that put them in the path of vehicles. Crossing height was strongly correlated with verge height, suggesting that elevated verges may have some value in mitigation, but increased flight height may be at the cost of reduced permeability. Green bridges should be explored as an alternative form of mitigation. Robust monitoring is essential to assess objectively the case for mitigation and to ensure effective mitigation.

## Introduction

Recent research shows that major roads can have a major negative impact on bats. Bat density and diversity have been found to decline in proximity to a major road, with the scale of the impact indicating a barrier effect [1]. Studies of *Myotis bechsteinii* also provide evidence for a barrier effect with contracted foraging areas and reduced reproductive success of bats roosting close to roads [2]. Road avoidance behaviour has been observed in commuting bats [3], and street lighting [4] and traffic noise [5] reduce foraging activity and success. Numerous studies show that bats are killed on roads by collision with vehicles e.g. [6], [7], [8], [9]. Roadkill is hard to quantify due to the difficulty of finding carcasses [10], but low reproductive rates make bats particularly vulnerable to elevated adult mortality e.g. [11].

Roads are detrimental to a wide range of animals, reviewed by [12], [13]. Crossing structures, both under and over roads, have been built in an attempt to maintain connectivity across the landscape, and numerous studies report use of these structures by mammals and reptiles e.g. [14], [15], [16], [17]. However, the use of a mitigation feature, the widely accepted criterion for success, does not make it effective. To be effective it must play a significant role in maintaining local population sizes. Evidence for a small, unspecified proportion of individuals using a structure to cross a road is not evidence for effective mitigation if a greater proportion crosses the road unsafely, is forced to reduce home range size or has to make longer journeys to find an alternative route. Reviews of mitigation techniques for a wide range of animals report that studies assessing use are typically qualitative [18], [19]. In one such review, only two out of 123 studies were able to conclude a positive effect of mitigation at the population level [20].

European bats are protected by EU and national laws, see [1]. Legally required mitigation measures on road developments usually take the form of crossing structures to maintain linear elements in the landscape that bats rely on for commuting. The use of wire bat bridges or ‘gantries’ is becoming increasingly common: at least eight have been built in the UK in the last five years and six more are planned for the A11 in Norfolk [21], [22]. However, there are no published data regarding the effectiveness of these structures. Recent reviews of case studies of bat mitigation in the UK found that most reports were at best qualitative and inconclusive [22], [23]. Green bridges, underpasses and culverts have been installed across Europe with potential use as a wildlife passage frequently being an unintended or secondary function. Most of the studies reporting their use by bats are unsuited to quantitative analysis, or fail to address the important distinction between use and effectiveness e.g. [24], [25], [26]. Seven bat species were caught flying through motorway underpasses in Germany, but when activity levels were compared with sites in the surrounding forest, only *Barbastella barbastellus* and *M. nattereri* were caught significantly more often in the underpasses, suggesting their effectiveness as crossing structures may be species-specific [2]. The use of underpasses by at least six bat species was also reported in Ireland, with the tendency to fly through the underpasses rather than over the road being related to the degree of clutter-adaptation of a species [27].

Our aim was to examine whether road crossing structures built for bats (or considered suitable for bats) are not only used but, moreover, are effective in guiding a significant proportion of bats safely over or under roads. The ideal study would determine the effectiveness of the structures in maintaining local bat population sizes, but this requires pre-construction data, which do not exist. We therefore studied their effectiveness in protecting crossing bats by reducing the risk of collision mortality. We studied underpasses, the most common wildlife crossing structure in Europe and North America, and wire bat gantries, bridge-like structures designed to guide echolocating bats over the road (see method for a detailed description). These are currently favoured in the UK and are also being built in other parts of Europe. They are sometimes referred to as bat bridges, but we have avoided this term to avoid confusion with other structures, such as green bridges.

## Materials and Methods

### Study Sites

**Figure 1A:**
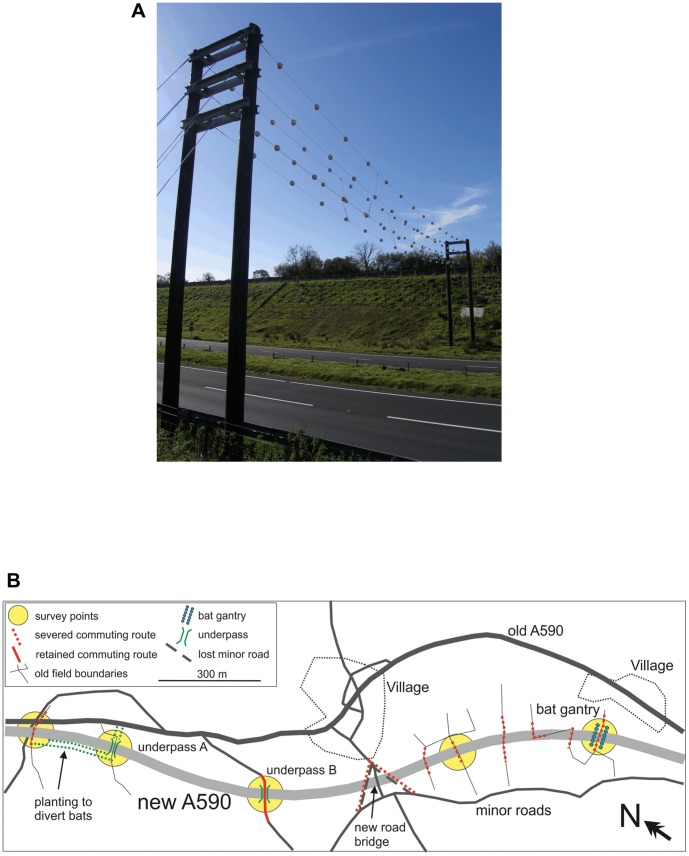
Photograph of a typical bat gantry (A590). **Figure 1B** **.** Map of the study sites on the A590.

All four study sites were located in northern England: three roads in Cumbria (A590 at 54°14′N, 2°55′W; A595 at 54°35.2′N, 3°33′W; A66 at 54° 38.3 N, 3°31.4′W), and one in Northumberland (A69 at 54°58′N, 2°15′W). All sites were located in rural lowland used primarily for agricultural grazing, with linear elements such as hedgerows, dry stone walls and tree lines providing connectivity for bats [28], [29]. The importance of all sites as bat foraging and commuting routes was established during pre-construction environmental impact assessment, see Appendix A in [22], but methodological differences and inadequate data in these assessments prevented comparison with this study. All roads were built to bypass nearby settlements with traffic volumes of 12,000–17,000 vehicles per day [30], [31]. All bat gantries were of similar design: two wooden or metal pylons erected at either side of the road with 2 or 3 pairs of wires spanning the road between them (approximately 20 m on two lane roads and 30 m on four lane roads), with plastic spheres at intervals of approximately 2 m, at a height of 6-9 m, and width of 2 m (see Fig. 1A for example). They are presumed to act as linear features that will guide echolocating bats across roads, above traffic height. At each gantry or underpass, we compared the number of bats using the structure with those crossing unsafely over the road. Where possible, we also compared crossing activity at the gantries and underpasses to that at adjacent or nearby severed but unmitigated commuting routes (as detailed below). The only sites we were unable to compare to nearby commuting routes were underpass B on the A590 and the A69 gantry, as explained below. Photographs are provided in Appendix S1.

The A590 High and Low Newton Bypass (Fig. 1B), opened in April 2008, is a 3.8 km dual carriageway (divided highway) in the Lake District National Park. Two underpasses, a bat gantry (Fig. 1A) and two severed but unmitigated pre-construction commuting routes (Appendix S1) were studied. Underpass A (30 m length ×6 m width ×3 m height) carries a bridleway (a wide footpath designed for horses, pedestrians and cyclists) beneath the road. It is located near to (but not on) a known commuting route, but trees and shrubs were planted along 200 m of the road in an attempt to divert bats from the unmitigated commuting route (on a severed tree-line to the north) that we also surveyed for comparison. Underpass B (30 m length ×6 m width ×5 m height, 500 m south of A) was built to carry a hedgerow-lined minor road, a known commuting route. There were no other previous commuting routes in close proximity for comparison to this underpass. The bat gantry, approximately 1000 m further south, crosses the road at a known commuting route along a severed hedgerow. We also surveyed a second unmitigated commuting route on a severed hedgerow 400 m north of the gantry, where the road now lies in a cutting (roadcut) up to 20 m deep.

The A595 Lillyhall to Parton Improvement, opened in December 2008, is a 5.1 km dual carriageway. A bat gantry was installed where the bypass bisected woodland (Appendix S1). We surveyed this gantry and an unmitigated commuting route 90 m to the north where a hedgerow was severed by the road.

The A66 Stainburn and Great Clifton Bypass, opened in December 2002, is a 4.2 km three-lane carriageway, bisecting a 30 m wide strip of mature woodland. The gantry is located at the western edge of the wood 15 m from the pre-construction commuting route, a bridleway within the wood. We surveyed both the gantry and the pre-construction commuting route. An underpass (15 m length ×5 m width ×2.5 m height) was built at the eastern edge of the wood to carry a small stream, and its use by crossing bats was also investigated.

The A69 Haydon Bridge Bypass, opened in March 2009, is a 2.9 km two-lane carriageway. A bat gantry was constructed at the site of a bat flight line where the road severed a hedgerow (Appendix S1). In pre-construction surveys, minimal bat activity was recorded on all potential commuting routes within 1 km, with the exception of Gee's Wood 800 m east, where the new road bridged a 100 m wide, 10–20 m deep wooded valley with a stream. We therefore conducted surveys at the gantry only.

### Survey Methods

Surveys were conducted in June and July, on the A590 in 2010, and all other sites in 2011. Ten 90 min surveys were completed at each crossing point, five commencing at sunset and five starting 90 min before sunrise. Only the five dusk surveys were conducted on the A69 due to low activity levels at dawn. Surveys were conducted on warm, still, dry nights to avoid weather dependent variation in bat activity. At each crossing point an observer was positioned on the verge (grassed bank) either side of the road, equipped with a Pettersson D240× broadband bat detector (http://www.batsound.com, Uppsala, Sweden) and a solid state recorder (Edirol R-09HR, http://www.roland.co.uk, Swansea, UK) set up to automatically detect and record bat echolocation calls (see [1] for details). Since all events were “time-stamped", observations of crossing bats were later matched to echolocation call recordings for species identification. A Pettersson D500× (http://www.batsound.com) bat detector (suitable for automated logging) was also placed in the central reservation (median) when one was present to increase the chances of detection and aid species identification. Both detectors provide recordings that preserve all essential frequency and amplitude information of the echolocation calls, making them the most appropriate choice for species identification. Bats recorded but not observed were excluded, although this was rare. Two observers were used to maximise observations and ensure crossing bats were not missed. All equipment was time synchronised and observers conferred via two way radios. Flight height, direction, distance from the gantry and time of crossing were recorded for each bat. Records were later combined and duplicates removed. Measured points of reference were used to estimate heights and distances to the nearest metre. The bat gantry, fencing and road signs were used for vertical references, and road markings and crash barrier posts provided horizontal reference points. A clear point of reference was always in view and estimations were made without difficulty. Flight height was recorded over the road, with the majority of bats (87%) crossing at constant heights. For those bats which altered their flight height during crossing (8% decreased height and 4% increased) the lowest flight height over the road was recorded. To corroborate observations night vision digital video cameras (Sony Nightshot DCR-SR75E and DCR-SR35, http://www.sony.co.uk, Basingstoke, UK) were set up on each verge facing the gantry or commuting route over the road, alongside heterodyne bat detectors used to indicate presence on the recordings (Batbox III, www.batbox.com, Steyning, UK) and infrared lights. However, these were found to be unnecessary with visual observations providing sufficient information.

At the underpasses, these methods were repeated on the road above and an additional observer with the same equipment was positioned at one end of the underpass below. Infrared lights were used to illuminate the underpass, and a night scope (Dedal generation 2, www.nightvision.ru) was used to aid observations. A Pettersson D500× bat detector (http://www.batsound.com) was placed in the centre of the underpass to aid in species identification.

### Species Identification

Batsound Pro software (http://www.batsound.com) was used to identify species from sonograms of their calls [32]. In most cases, *Myotis* and *Nyctalus* were identified only to genus because of similarity in call structure [32]. *Myotis nattereri*, *M. mystacinus*, *M. daubentonii* and *M. brandtii* are widespread in the area [33]. *Nyctalus noctula* is widespread, and *N. leisleri* is rare. However, *Nyctalus* data were not analysed as bats flew at heights greater than 15 m over the road and commuting activity was low at most sites. A small proportion (<5%) of *Pipistrellus* calls was classified only to genus level, because of the overlap of call parameters of *P. pipistrellus* and *P. pygmaeus*. *Plecotus auritus* was also present, but will have been under-recorded because of its low intensity echolocation call [32]. Species identification was not reliable for 30% of crossing bats due to noise or low intensity recordings. These records were therefore omitted for species specific analyses. All records (excluding *Nyctalus*) were used in all other analyses.

### Definitions

‘Safe’ and ‘unsafe’ crossing heights were defined as being greater and less than 5 m above the road surface respectively. The maximum height for heavy goods vehicles in the UK is 4.9 m [31]. Bats crossing the road below 5 m are therefore at risk of collision.

Two estimates of ‘use’ of the gantry were defined: bats crossing the road within 2 m or 5 m of the gantry at a safe height. These definitions are based on observations from the literature: *Myotis mystacinus* commuting at dusk from a roost to a foraging area flew 0.3–1.7 m from a hedgerow, with the greatest distances recorded only at irregularities in the hedge structure [34]. Commuting *M. daubentonii* flew 3.2–5.8 m from a forest edge and 2.1–4.5 m from a wall [35].

### Data Analysis

We have provided statistical analyses, but in some cases, whether or not a particular result was statistically significant contributed little towards assessing the effectiveness of the crossing structures. For example, even if significantly more bats cross a road safely than unsafely, the impact on population trends ultimately depends on the proportion of the population that is killed in collisions. Statistical analyses were carried out using R [36]. Wilcoxon signed ranks tests used the function *Wilcoxsign_test* from the package *coin* [37] to compare activity per survey (n = 5 for A69, n = 10 for all other sites) between underpasses and the road above and between bats crossing at gantries and at unsafe heights below. Each survey was treated as independent. Although activity was generally lower at dusk, there was no observable variation in the behaviour of crossing bats between dusk and dawn and so the data were combined. Some individuals may have been recorded several times during surveys but this was unavoidable, and each crossing event was considered to be important regardless of this. The relationship between flight height and verge height was investigated using Spearman's rank correlation (*cor.test*) and comparisons were made between species using Kruskal–Wallis tests (*kruskal.test*) and pairwise Wilcoxon rank sum W tests (*wilcox_test* function, package *coin*) with Bonferroni corrections. On the A66, observations were made across the entire 30 m section of severed woodland that included both the gantry and the severed commuting route. The heights and positions of all crossing bats were used to generate a kernel estimate of crossing intensity, using the *density* function in the *spatstat* package [38].

## Results

### Underpasses

**Figure 2:**
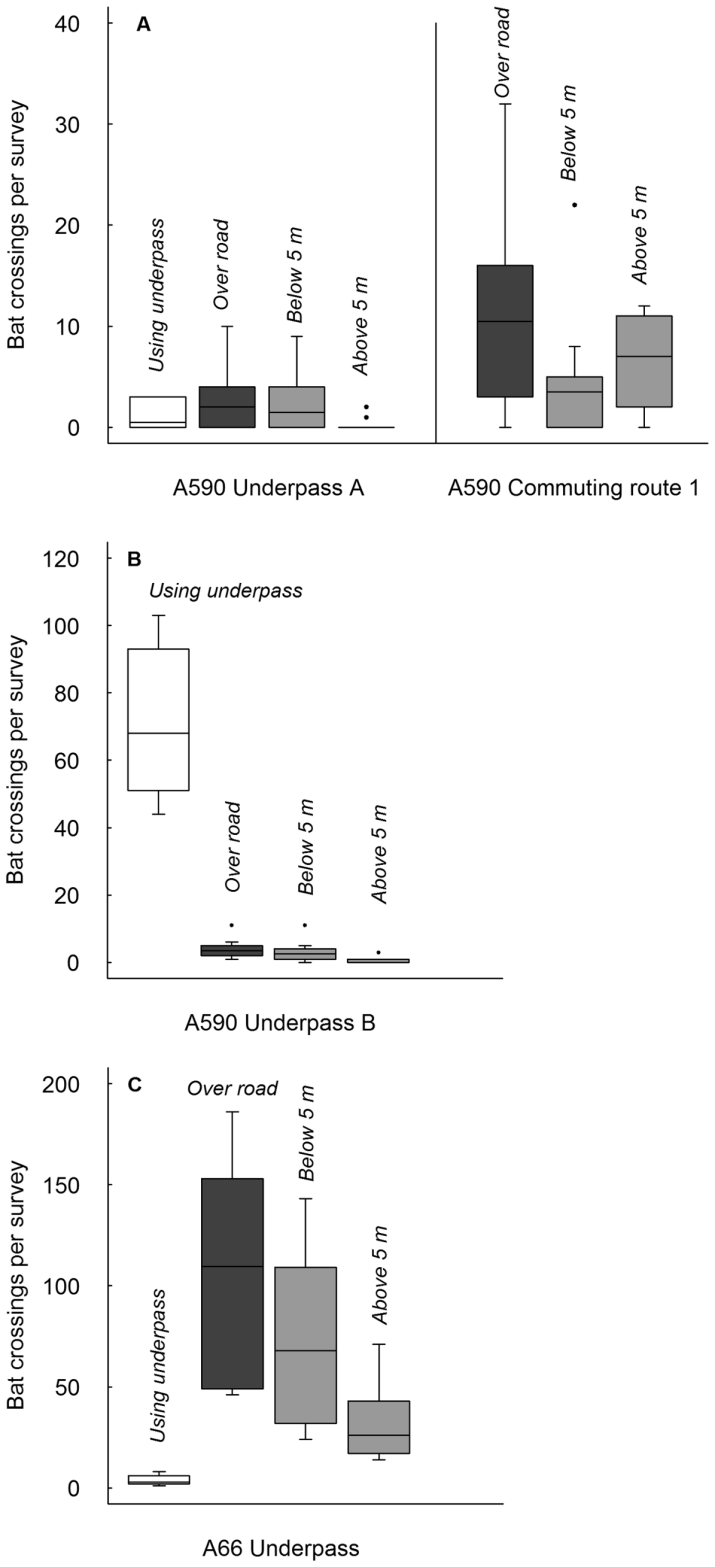
Boxplots of the number of bats crossing per survey at each underpass. Boxplots (median with upper and lower quartiles) for the number of bats crossing per survey (n = 10) at each underpass (numbers crossing using underpass, over the road above and at safe and unsafe heights over the road), and at the unmitigated commuting route on the A590 which was diverted to underpass A (numbers crossing over the road and at safe and unsafe heights).

At underpass A on the A590, activity was low (Table 1, Fig. 2A), but 69% bats preferred to fly over the road rather than use the underpass (Z = −2.39, *P* = 0.03). Of bats crossing the road, 88% did so at unsafe heights. *Pipistrellus pipistrellus* and *P. pygmaeus* were detected in the underpass. *P. pipistrellus*, *Myotis* and *Plecotus auritus* were detected flying over the road. Over the same period, more bats crossed the road at the nearby unmitigated, severed commuting route (Table 1, Fig. 2A), 58% at unsafe heights. Most were *P. pipistrellus* and *P. pygmaeus*, approximately half crossing below 5 m. Four of the five *Myotis* detected crossed below 5 m. No bats were observed flying along the planted diversion to the underpass, but observers were only able to monitor this where it left the original commuting route.

**Table 1:** The crossing behaviour of all bats from all surveys for each study site (NB the number of bats crossing at safe heights at the gantries includes those ‘using’ the gantry).

Road	Site	Total crossing	‘Using’ gantry (within 2 m)	‘Using’ gantry (within 5 m)	Using underpass	Unsafe height over road	Safe height over road
A590	Underpass A	36	-	-	11	22	3
	Commuting route 1	113	-	-	-	65	48
	Underpass B	904	-	-	864	32	8
	Bat gantry	104	11	31	-	43	61
	Commuting route 2	19	-	-	-	0	19
A595	Bat gantry	96	1	6	-	81	15
	Commuting route	77	-	-	-	72	5
A66	Combined survey area	1117	24	100	39	751	327
	(Bat gantry, Commuting						
	route & underpass)						
A69	Bat gantry	65	5	27	-	10	55

Activity levels were higher at underpass B than at A (Table 1, Fig. 2B) and many more bats (96%) used the underpass than flew over the road above at unsafe heights (Z = 2.80, *P* = 0.002). *P. pipistrellus*, *P. pygmaeus* and *Myotis* were all detected both in the underpass and over the road.

Activity in the underpass below the A66 was low (Table 1, Fig. 2C), with only 4% of bats crossing through it, in comparison to 60% crossing at unsafe heights over the road above (Z = 2.80, *P* = 0.002). *P. pipistrellus*, *P. pygmaeus* and *Myotis* were detected over the road and in the underpass.

### Bat Gantries

**Figure 3:**
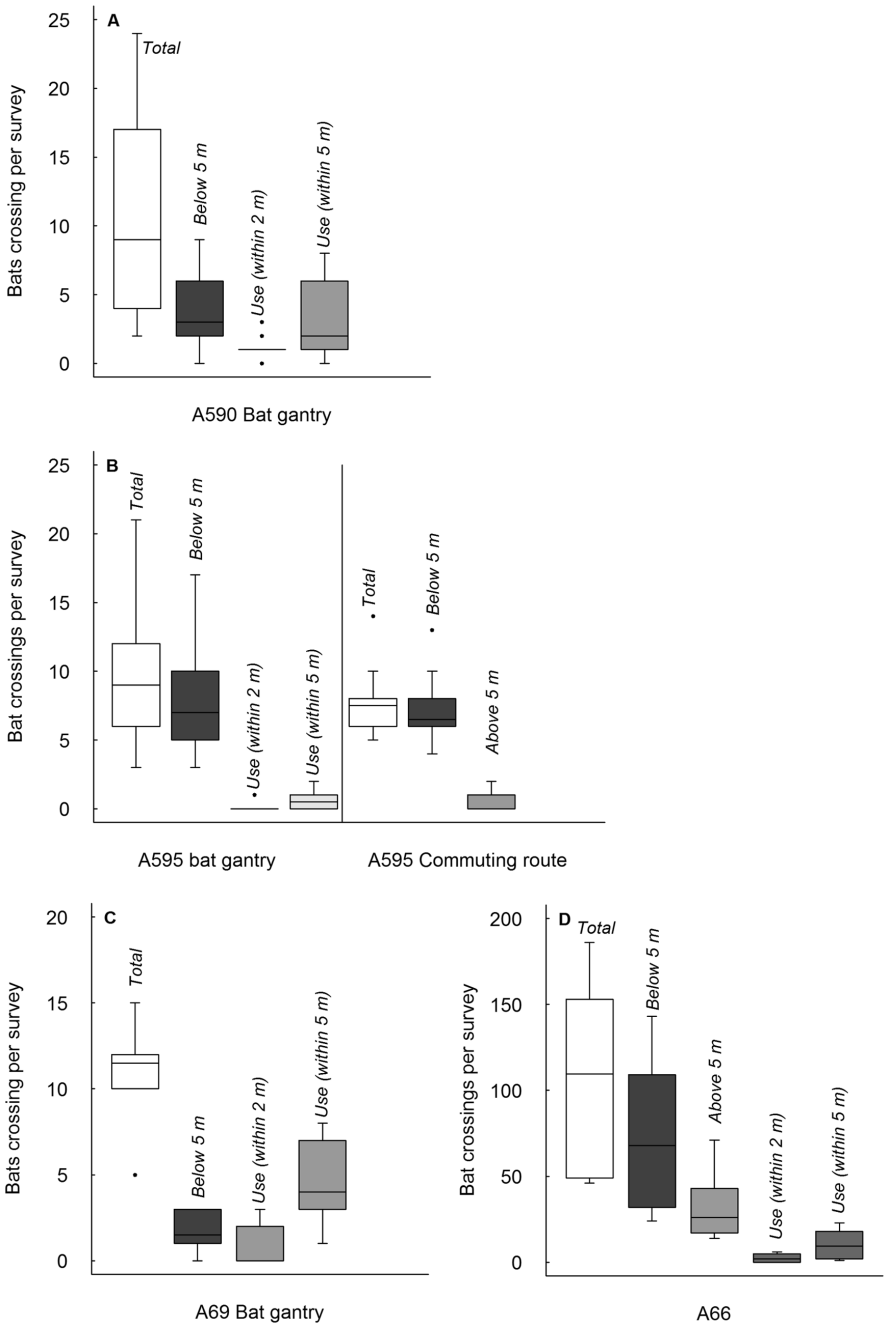
Boxplots of the number of bats crossing per survey at each bat gantry. Boxplots (median with upper and lower quartiles) of the number of bats crossing per survey (n = 10) at the four bat gantries, together with data on total number crossing, the numbers crossing at safe and unsafe heights, numbers ‘using’ the gantry according to both estimates (within 2 and 5 m), and the numbers crossing at nearby, unmitigated, severed commuting route nearby.

At all sites, few bats crossed using the gantry (Fig. 3). At the A590 gantry, four times as many (41%) crossed the road at unsafe heights as crossed within 2 m of the gantry (11%; Z = 2.61, *P* = 0.008), and 1.4 times as many as crossed within 5 m of the gantry (30%; Z = 1.49, *P* = 0.15). At the A595 gantry (Fig. 3B), far more bats (84%) crossed the road at unsafe heights than flew within 2 m (<1%; Z = 2.81, *P* = 0.002) or 5 m (6%; Z = 2.81, *P* = 0.002) of the gantry. At the A69 gantry (Fig. 3C), more bats crossed the road at unsafe heights (17%) than flew within 2 m of the gantry (8%; Z = 1.17, *P* = 0.31), but fewer bats crossed at unsafe heights compared to those flying within 5 m of the gantry (42%; Z = −2.14, *P* = 0.06).

**Figure 4:**
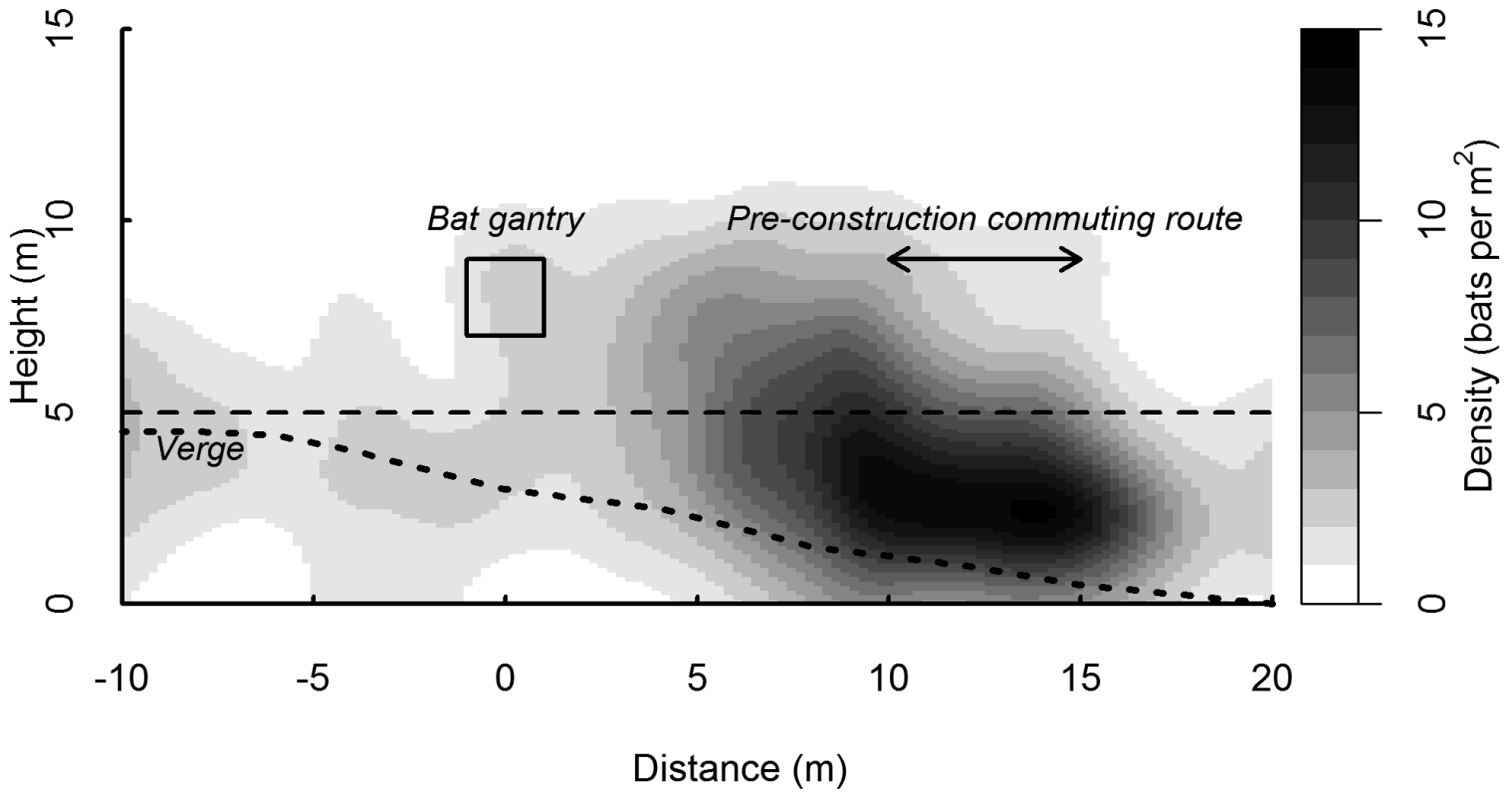
Kernel intensity estimation of the density of crossing bats across the A66 site. Gaussian kernel and bandwidth of 1 m used (n = 1078). The section of severed woodland at the A66 site is shown. The gantry is located at distance 0 m (height marked by square), and the pre-construction commuting route at 10–15 m. ‘Unsafe’ crossing heights are located below the dashed line. The dotted line marked verge shows the decrease in verge height above the road from left to right.

At the A66 survey area (including both the gantry and the pre-construction commuting route, Fig. 3D), far more bats (70%) crossed at unsafe heights, than flew within 2 m (2%; Z = 2.81, *P* = 0.002) or 5 m (9%; Z = 2.81, *P* = 0.002) of the gantry. The kernel density estimation for the A66 (Fig. 4) shows a high concentration of bats crossing at unsafe heights centred at the unmitigated pre-construction commuting route, and low activity around the gantry.

On the A595 the number of bats crossing at the nearby unmitigated, severed commuting route (Fig. 3) was comparable with that crossing in the vicinity of the gantry, and 94% of bats crossed at unsafe heights.

At the unmitigated, severed commuting route near the gantry on the A590 foraging activity of *P. pipistrellus* was observed during all surveys on the western side of the road, but only 19 bats crossed over 10 surveys, all at safe heights. Although other severed commuting routes (shown in Fig. 1) were not surveyed, significant crossing activity was not observed during reconnaissance.

### The Influence of Verge Height

**Figure 5:**
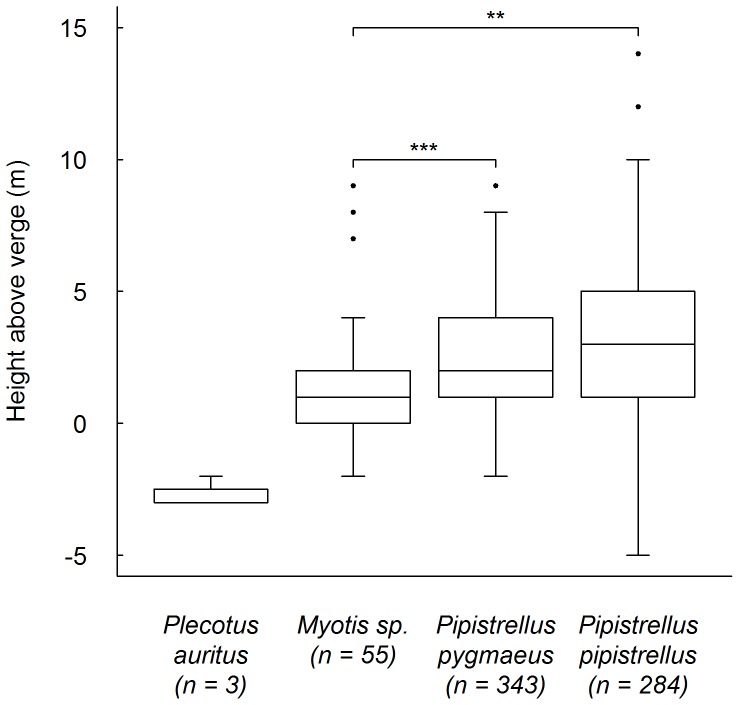
Boxplot of flight height above verge height of identified crossing bats at all sites. Median with upper and lower quartiles. Significant differences shown for *Myotis* and *Pipistrellus* species ** *P*<0.0005, *** *P*<0.0001. Excludes underpass sites. Verges are elevated on either side of the road and are above road height, therefore negative values indicate bats flying across the road below the height of the verge.

The mean crossing height of all bats across all sites (excluding underpasses) was positively correlated with verge height (estimated to nearest 0.5 m) at the point of crossing (Spearman's rank; r = 0.34, n = 1552, *P*<0.0001). This correlation was significant at the species/genus level, with *Myotis* showing the strongest relationship (*Myotis*: r = 0.46, n = 55, *P*<0.001; *P. pipistrellus*: r = 0.40, n = 284, *P*<0.0001; *P. pygmaeus*: r = 0.34, n = 343, *P*<0.0001). Crossing height above the height of the verge was found to vary between genera (Fig. 5). No difference was found between the two *Pipistrellus* species (Wilcoxon rank sum; W = 47193.5, *P*>0.05 after correction), but *Myotis* flew significantly lower than both *P. pipstrellus* (Wilcoxon rank sum; W = 5306.5, *P*<0.0005 after correction) and *P. pygmaeus* (Wilcoxon rank sum; W = 5935, *P*<0.0001 after correction). Only three *P. auritus* were detected, and all crossed below the height of the verge at <3 m over the road.

## Discussion

This is the first study to assess the effectiveness of road crossing structures for bats, by measuring the proportion of individuals that used these structures to cross safely. Although a limited study of such diverse structures cannot be definitive, we believe it demonstrates that some current practices are failing. We found no evidence that bats used gantries in preference to nearby, severed but unmitigated commuting routes. At all but one site (A69, where activity was low), the majority of bats crossed at unsafe heights, even in proximity to gantries. Of seven mitigation structures studied, only one underpass was effective in carrying the majority of bats safely across the road.

### Underpasses

Underpass A on the A590, and the A66 underpass, are not effective mitigation measures: very few bats flew through them relative to the number crossing at unsafe heights over the road above, and in the case of underpass A, at an original commuting route nearby. Underpass B on the A590 showed high levels of use by commuting bats, with just 4% crossing at risk of collision mortality on the road above. This underpass is effective in allowing bats to cross the road safely. However, the lack of robust pre-construction population data makes it difficult to assess how effectively this underpass can protect bat populations. Even though a high proportion of bats use the underpass, if bat populations have declined since construction and the road acts as a barrier, then the underpass becomes ineffective. Nevertheless, underpass B preserved a pre-construction commuting route, with no necessity for commuting bats to alter their flight course or height. Although replication is needed, this shows that underpasses can be effective when built over existing commuting routes. This makes sense in the context of the high fidelity that bats show to their commuting routes [39], [40], [41]. Underpass A and the A66 underpass were unsuccessful probably because they require commuting bats to alter their course and flight height. Both underpasses are also lower than underpass B, but several studies report bats flying through even smaller structures e.g. [24].

### Bat Gantries

Bats did not cross at gantries more than at unmitigated road crossings, and gantries did not effectively increase the height at which bats flew above the road. There was no evidence that bats were ‘using’ gantries by flying in close proximity to them, as they do along hedges [34], [35].

These bat gantries are failing to perform the function for which they were built, even at well-established sites such as the A66, where the gantry has been in place for nine years and is only 10–15 m from the original commuting route. Although road kill counts were not performed, it is well documented that bats are killed on roads in high numbers [6], [7], [8], [9] and mortality may be high enough to be unsustainable [23].

### Verge Height

The strong correlation between verge height and the average crossing height of bats suggests that increased verge height may have some potential in raising flight height above traffic. This effect was found to vary between species: *Myotis* species were most sensitive to changes in verge height and flew closer to the verge than *Pipistrellus* species, as did the few *P. auritus* observed. However, increased verge height generally widens the open terrain that must be crossed (since they are inclined away from the road, higher verges are usually wider, see photographs in Appendix S1), which could deter some species from crossing, increasing the barrier effect. Very few bats crossed the road at the second unmitigated commuting route on the A590, where verges are 20 m high, and the width of the open space is 80 m. Similarly, in Germany, *M. bechsteinii* were observed to frequently fly over a two lane road with a connecting tree canopy, but not over a four lane motorway with a gap in the forest [2].

### Habitat Continuity

It has been suggested that crossing structures will be more effective if continuous with the vegetation on either side of the road [8]. However, even though the A66 gantry is connected to mature woodland on either side and is only 10–15 m from the commuting route, it is still ineffective. Commuting bats use linear habitat elements not just for navigation, but also to obtain protection from predation and wind and as foraging microhabitats [29], [42]. More substantial structures that provide shelter and perhaps bear a closer resemblance to natural features are likely to be more successful, for example a planted green bridge that provides a continuation of hedgerow, or tree lines over the road. Green bridges, although built for other wildlife, are only just being considered as mitigation measures for bats, and evidence is still needed for their effectiveness. Ten species of bat were found to use green bridges in Germany, with higher use than conventional road bridges, but results focussed on bats using the structures and did not look at those crossing the road below [25]. A simpler (but as yet untested) alternative that may be practical and effective on narrower roads is the ‘hop-over’: mature trees that overhang the road so that their crowns bridge the gap above the road [43].

### Species – Specific Effects

*Nyctalus* species do not appear to be adversely affected by roads. High foraging activity was observed over traffic at one site (A69), and small numbers of commuting *Nyctalus* were observed over the A590 at heights of over 15 m above the road. In other studies *Nyctalus* species have been observed flying high over roads with no recordings in underpasses [1], [2], [24], and low incidences of collision mortality [6], [7]. Roads have also been found to have less of an impact on habitat use by species that also forage in more open habitat, such as *Barbastella barbastellus* [2].

All other species detected in this study crossed at unsafe heights over the road. Differences in crossing heights were found between species, with *Myotis* species (and the three detected *P. auritus*) flying lower over the road than *Pipistrellus* species, increasing their vulnerability to collision mortality.

### The Effectiveness of the Survey and Monitoring Process

The bat gantries and one of the underpasses were installed because they were believed to be on significant commuting routes. However, we found activity was low at all gantry sites with the exception of the A66. Either activity has greatly diminished post-construction, adding to the conclusion of mitigation failure, or the assessment of these sites as major commuting routes was perhaps flawed. The absence of robust pre-construction monitoring means that we cannot say which explanation is correct. This raises serious questions about the effectiveness of the survey, assessment, mitigation and monitoring process. Several of the structures we have shown to be ineffective were said to be working in the commissioned reports [22], using the criterion that bats were seen to use them. Are other aspects of the reports equally flawed? Limited resources are available for conservation and it is vital that they are used effectively. Failure to do so makes no contribution to conservation and alienates further those outside conservation who question the use of public funds on conservation, e.g. the recent spending of £0.5 M on bat gantries [44], [45].

### Conclusions and recommendations

We assessed only a small number of mitigation structures, but the results are sufficiently striking that wider appraisal is essential if mitigation against road construction is to be effective. Wire bat gantries, of the type studied, should not be used, and attempts to divert original commuting routes should, if possible, be avoided. Underpasses built on existing commuting routes can be effective crossing structures, if commuting bats can maintain their original course and flight height. Further investigation into more substantial, natural crossing structures over roads, such as green bridges, and simpler options such as tree ‘hop-overs’, is needed. Unique aspects of individual sites, such as tree cover, hedges and topography must be exploited to make mitigation solutions as natural as possible and appropriate to the bat species present. Robust and comparable pre- and post-construction monitoring must be carried out that assesses more objectively the need for mitigation and its effectiveness.

## Supporting Information

Appendix S1 **Photographs of study sites.**
(ZIP)
