# Supplementary material for: Do Bat Gantries and Underpasses Help Bats Cross Roads Safely?
Source: PLoS One. 2012 Jun 13;7(6):e38775. doi: 10.1371/journal.pone.0038775 (PMC3374807; doi:10.1371/journal.pone.0038775)
Supplement: Appendix S1 — Photographs of study sites. (ZIP) [file pone.0038775.s001.zip › Berthinussen & Altringham App S1 revised.docx]

**Supporting Information. Berthinussen and Altringham 2012**

**Appendix S1.** Photographs of study sites


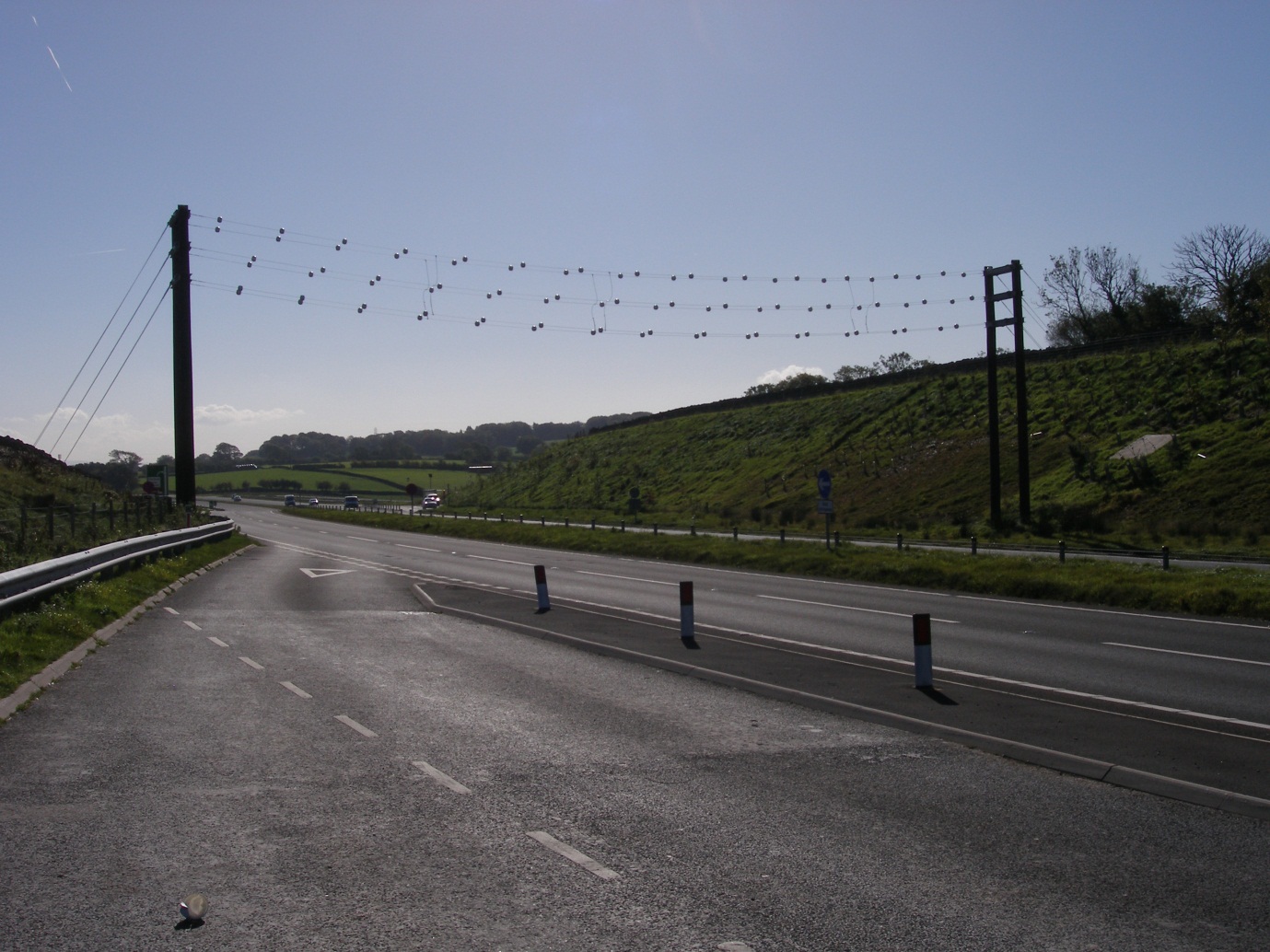


**Verges (banks)**

**Bat gantry**

**Central reservation (median)**

A590 bat gantry, taken from the south-east, with features labelled.


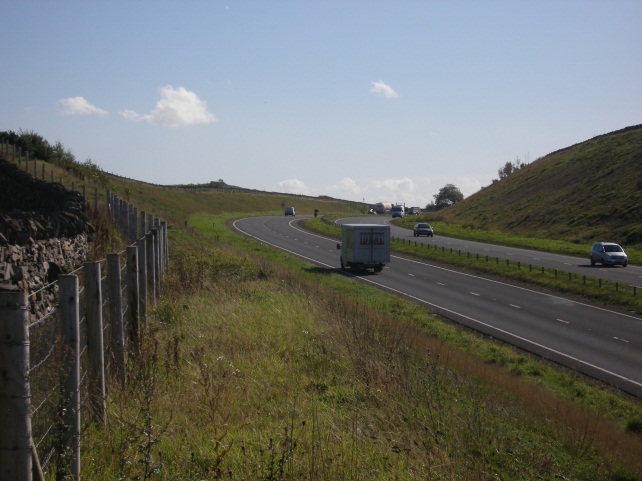

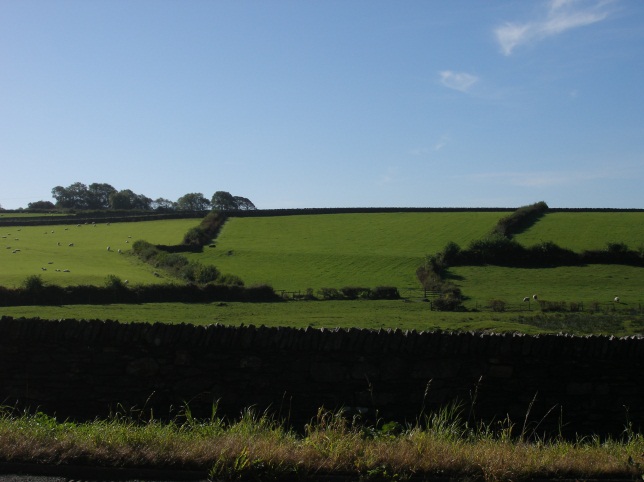


A590 unmitigated severed commuting route near the gantry (left – view of the severed commuting route marked in red from the north-east; right – hedgerows on the east severed by the road in cutting marked by arrows).


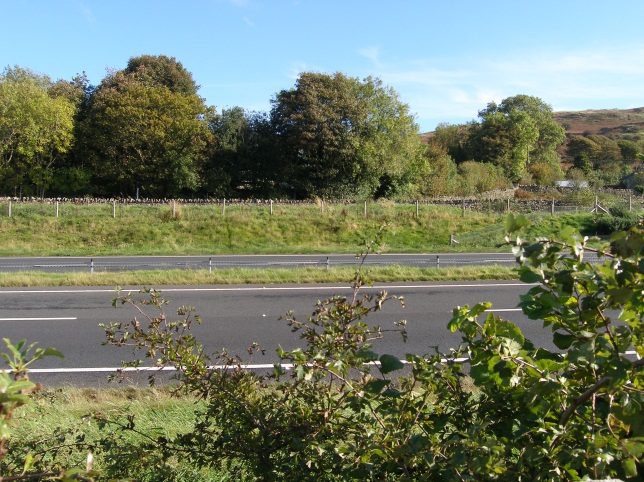

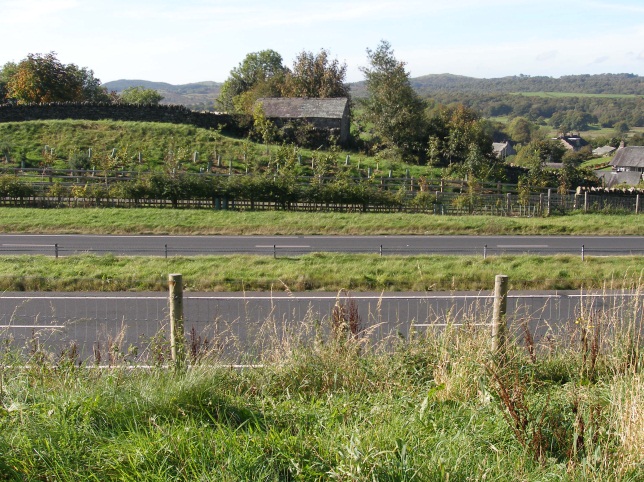


A590 unmitigated commuting route (marked in red) near underpass A (left – looking across from the west to the severed tree line, right – looking across from the east).


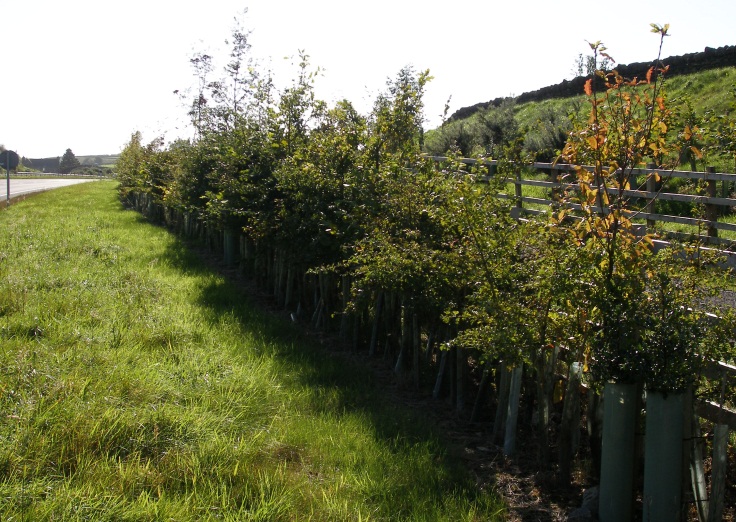


Trees and shrubs (approximately two years after planting) put in to divert bats from the commuting route on the A590 to Underpass A, taken on the western verge looking south.


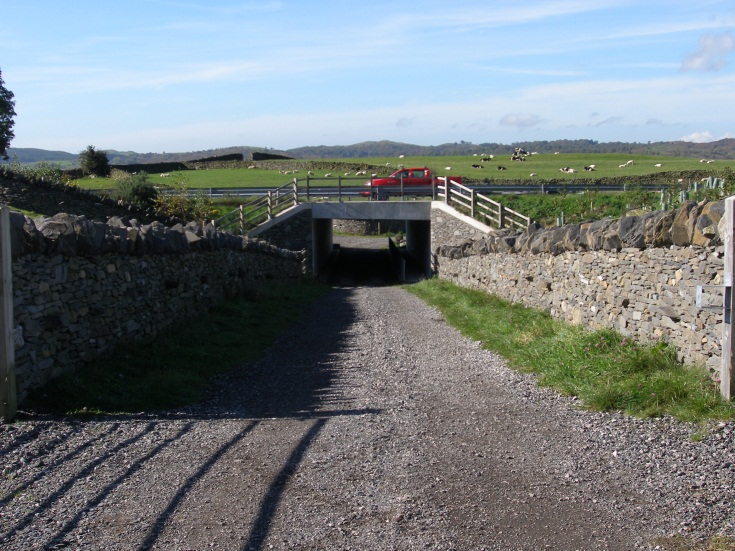

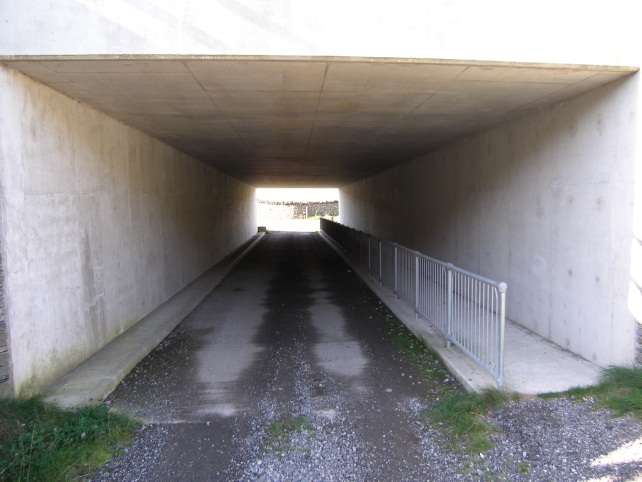


A590 Underpass A (both photographs from the east).


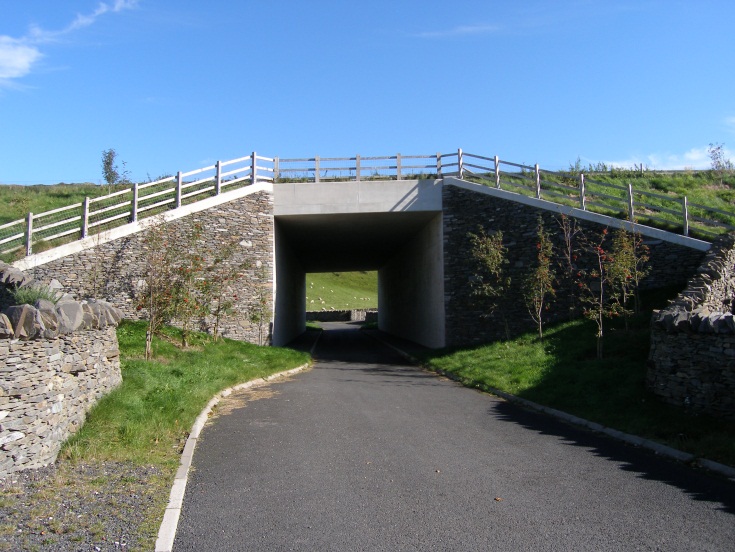

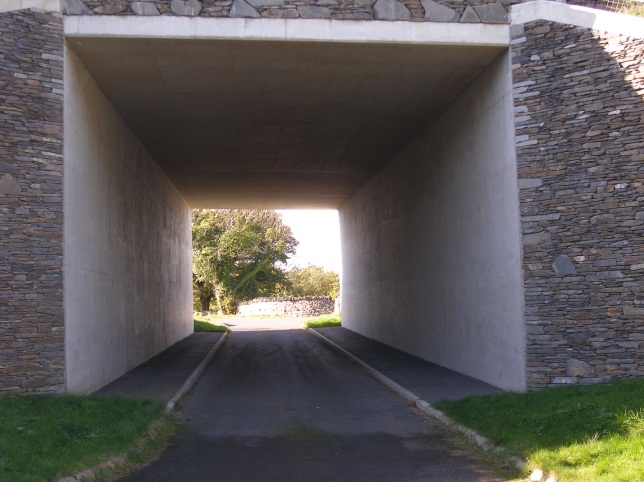


A590 Underpass B (left –from the west, right –from the east)


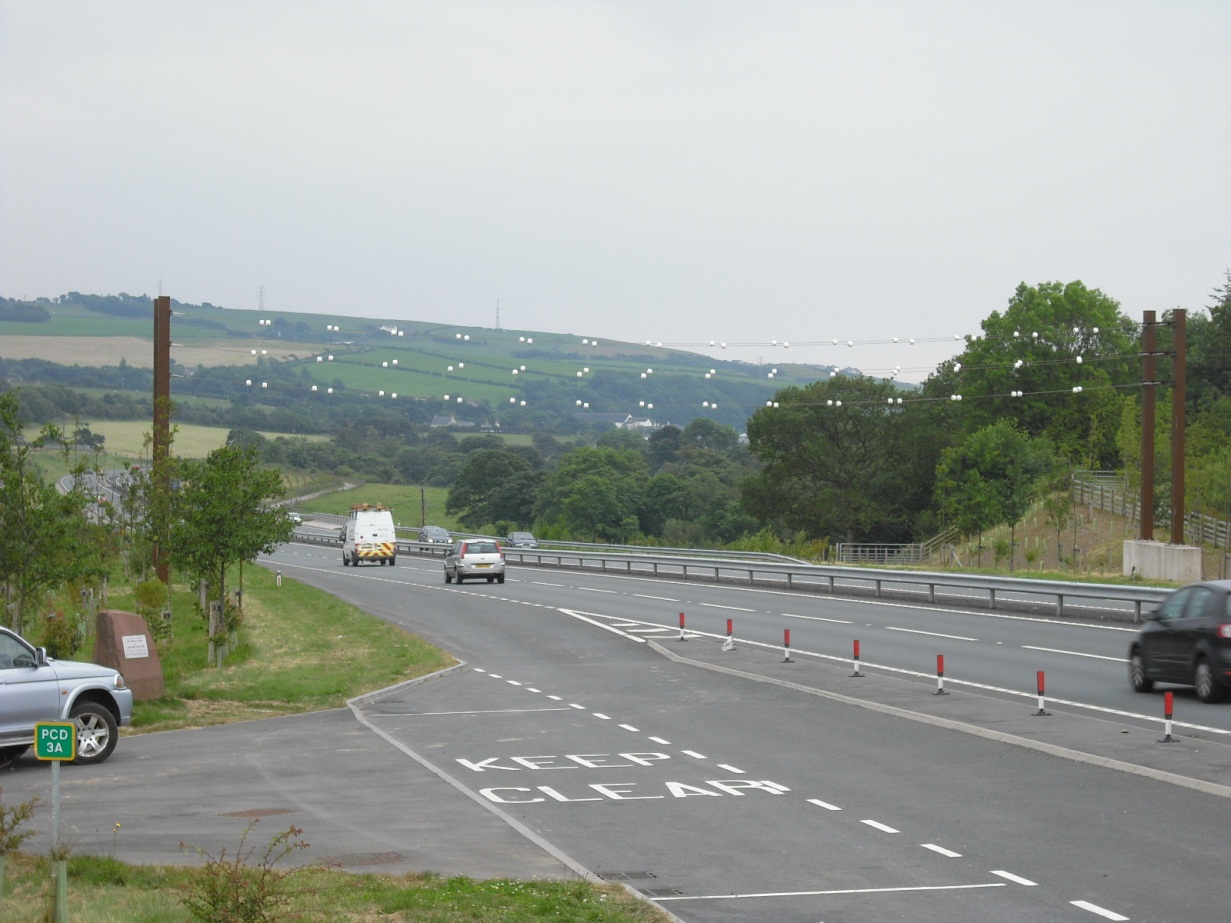


A595 bat gantry from the north-east.


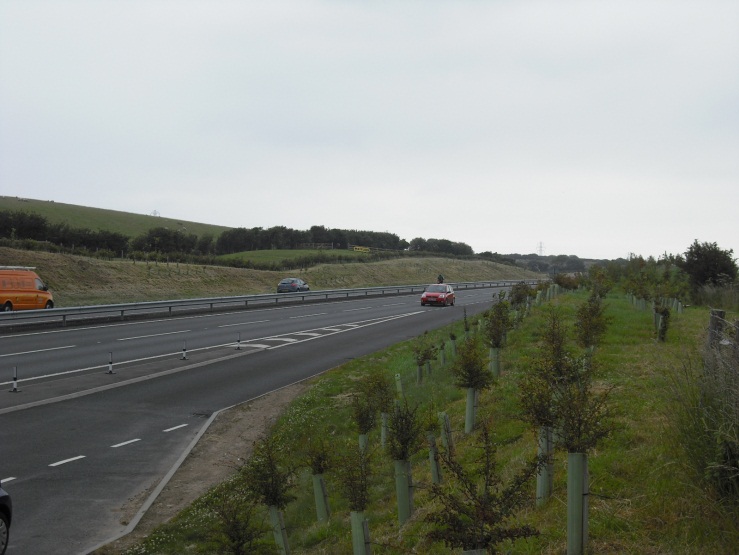

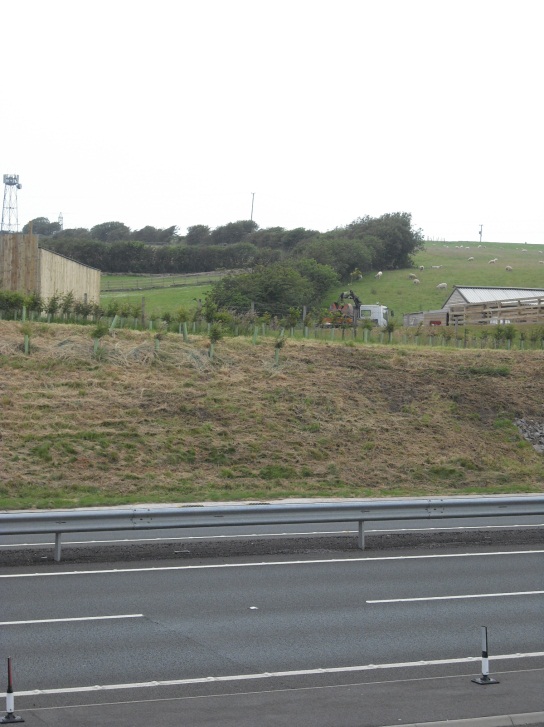


A595 unmitigated commuting route marked in red (left - from the south west, right – from the west side to the severed hedgerow).


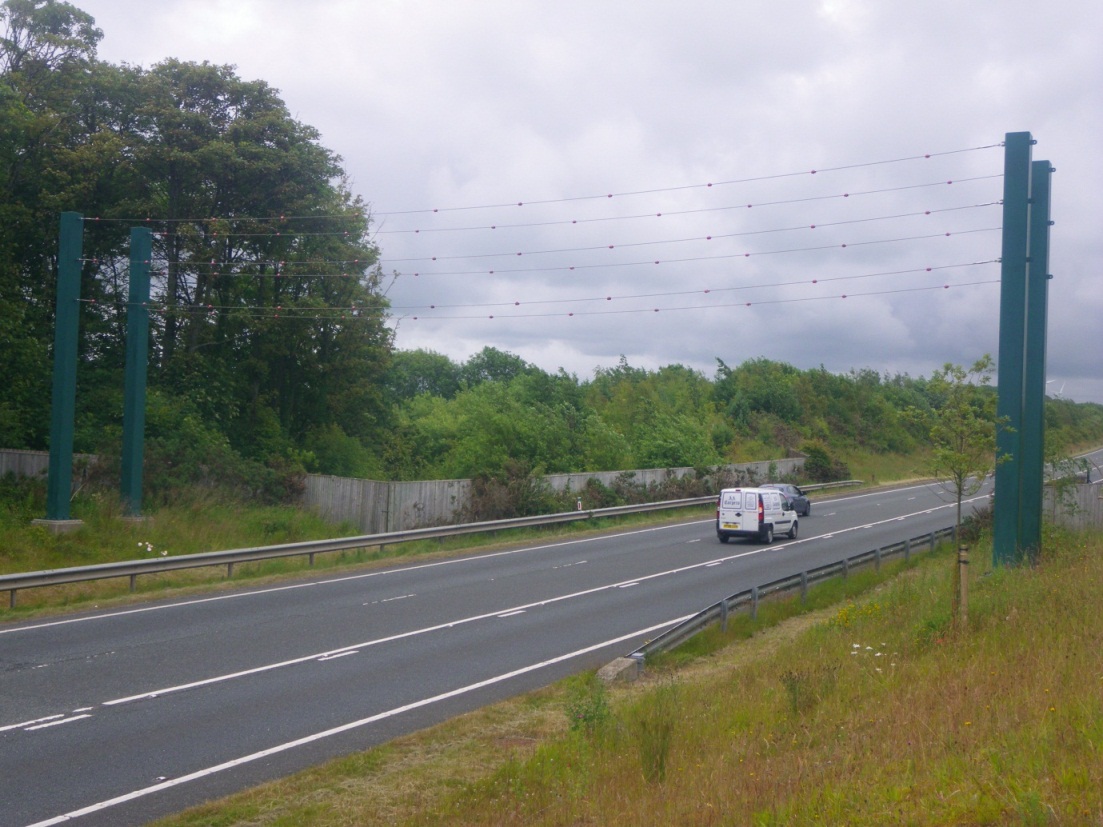


A66 bat gantry from the south-west.


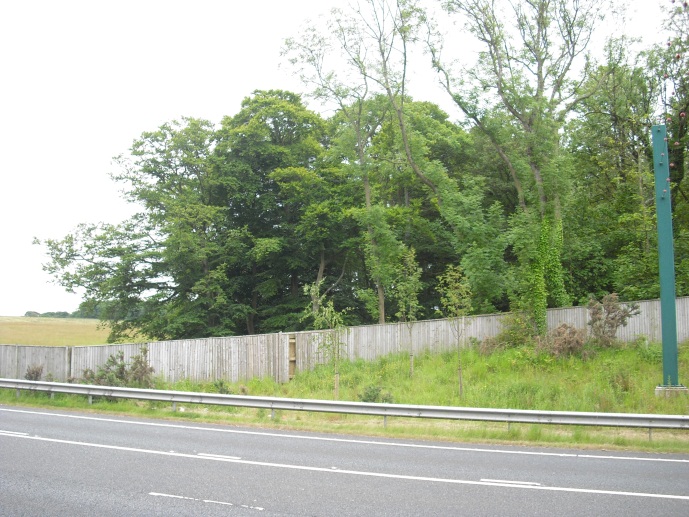

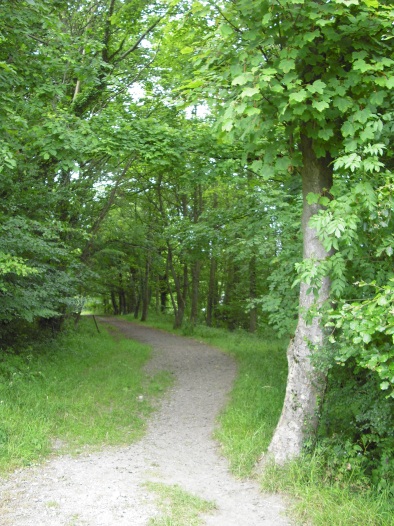


A66 unmitigated commuting route (left –from the north, commuting route marked in red, right – the bridleway of the original commuting route severed by the road, north of road).


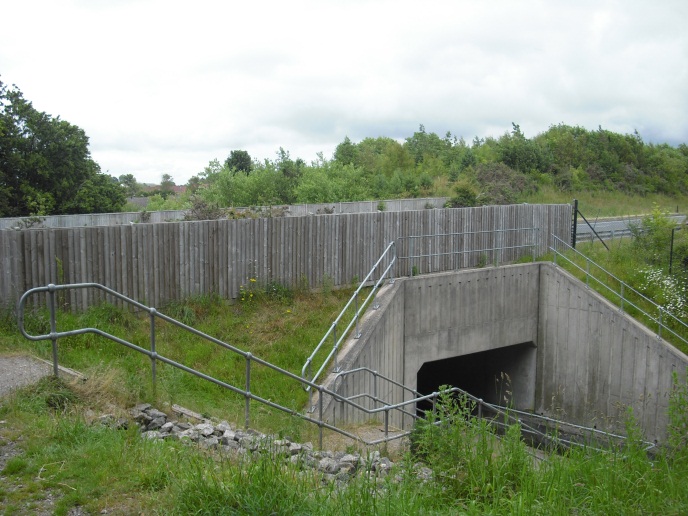

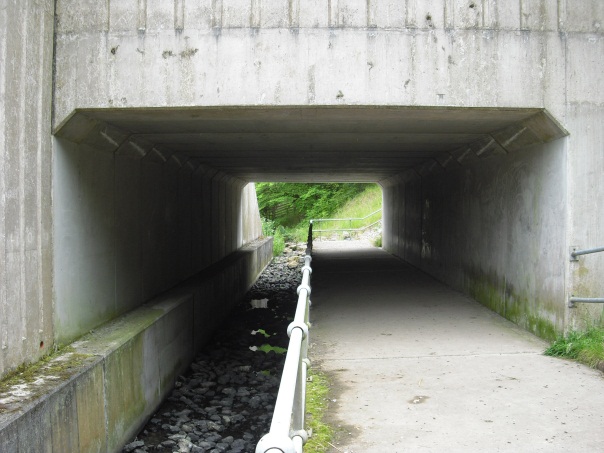


A66 underpass (left – taken from the south side at road level, right- taken from the north at underpass level).


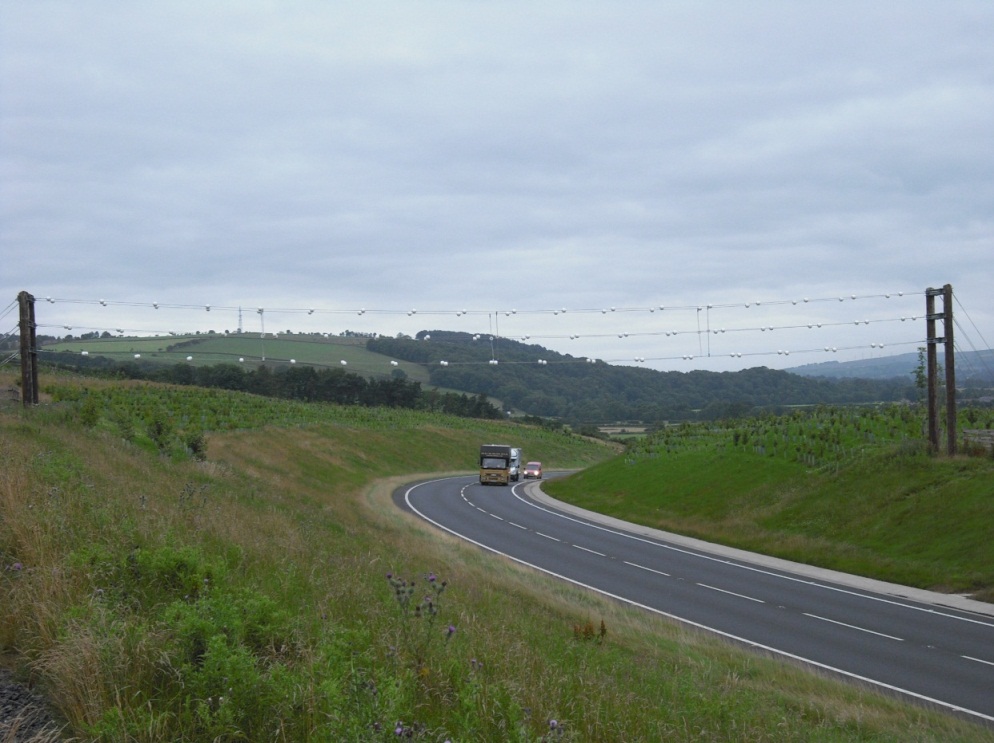


A69 bat gantry, from the south verge looking west.
